# Is there a change in P300 evoked potential after 6 months in cochlear implant users?

**DOI:** 10.1016/j.bjorl.2021.10.002

**Published:** 2021-11-10

**Authors:** Maria Stella Arantes do Amaral, Victor G. Calderaro, Henrique Furlan Pauna, Eduardo T. Massuda, Ana Cláudia. M.B. Reis, Miguel Angelo Hyppolito

**Affiliations:** a Universidade de São Paulo, Faculdade de Medicina de Ribeirão Preto, Departamento de Oftalmologia, Otorrinolaringologia e Cirurgia de Cabeça e Pescoço, Ribeirão Preto, SP, Brazil; b Universidade de São Paulo, Faculdade de Medicina de Ribeirão Preto, Departamento de Ciências da Saúde, Ribeirão Preto, SP, Brazil; c Universidade de São Paulo, Faculdade de Medicina de Ribeirão Preto, Hospital das Clínicas, Ribeirão Preto, SP, Brazil; d Universidade de São Paulo, Faculdade de Medicina de Ribeirão Preto, Ribeirão Preto, SP, Brazil

**Keywords:** Cochlear implantation, Event-related potentials, P300, Hearing loss

## Abstract

**Objective:**

There are few studies on long-latency auditory evoked potential (P300) in people with hearing loss who use a cochlear implant. Central auditory system evaluation with behavioral and electrophysiological tests is believed to help understand the neuroplasticity mechanisms involved in auditory functioning after cochlear implant surgery. This study investigated the electrophysiological processing of cortical level acoustic signals in a group of 21 adult individuals with postlingual bilateral severe-to-profound hearing loss who were submitted to cochlear implant surgery.

**Methods:**

Data were collected in three phases: pre-cochlear implant surgery, at cochlear implant activation, and 6 months after surgery. P300 measures were also registered during all phases. Tone-burst and speech stimuli were used to elicit P300 and were presented in free field.

**Results:**

Mean P3 component latency with tone-burst and speech stimuli were 352.9 and 321.9 ms in the pre-cochlear implant phase, 364.9 and 368.7 ms in the activation phase, 336.2 and 343.6 ms 6 months after the surgery. The P3 component mean latency values using tone-burst at activation were significantly different from those 6 months after cochlear implant. They were also significantly different using speech, between pre-cochlear implant and activation phases. Lower P3 component latency occurred 6 months after cochlear implant activation with tone-burst and pre-cochlear implant with speech stimulus. There was a weak correlation between mean P3 component latency with speech stimulus and time of hearing loss. There was no difference in amplitude between phases or in the comparison with the other variables.

**Conclusion:**

There were changes in P3 component latency during the period assessed, for both speech and pure-tone stimuli, with increased latency in the activation phase and similar lower results in the two other phases, Pre-CI and 6 months after CI use. Mean amplitude measures did not vary in the three phases.

## Highlights

• The lowest latency of P3 occurred six months after CI-activation for the tone burst stimulus.
• The lowest latency of P3 occurred Pre-CI with speaking stimulus.
• There was a poor correlation between the mean latency of P3 and the time of hearing loss for the speech stimulus.
• There was no variation of the P3 amplitude after 6 months using cochlear implant.
• There was an increase of P3 latency in individuals older than 45 years, for both puretone and speech stimuli.

## Introduction

P300 is a long-latency potential, evoked by auditory, visual, or somatosensory stimuli, capable of providing information about stimulus-related cortical processing response time. The P3 endogenous component is influenced by the brain's functional use represented by a positive wave occurring about 300 ms after stimulus onset.^1^ Since it can provide information on cortical processing time and brain response to stimuli, P3 component has been one of the most studied P300 components.

Regarding Cochlear Implant (CI) users, P300 may be useful to assess cognitive aspects,^2^, ^3^, ^4^ predict the success of the CI surgery,^5^ and elucidate post-CI surgery changes in auditory perception.^6^

Studies in the published literature have used speech stimulus^5^, ^7^, ^8^, ^9^, ^10^, ^11^ and pure-tone to elicit P300 in adult CI users.^3^, ^12^, ^13^, ^14^, ^15^, ^16^

Regarding their results, Micco et al. (1995)^5^ did not find any significant differences in P3 component amplitude and latency when speech stimulus was used, comparing CI users with normal hearing individuals. However, other authors showed diverging results for both speech and pure-tone, most of them indicating increased P3 component latency and decreased P3 component amplitude in CI users when compared with normal hearing subjects.^7^, ^9^, ^10^, ^11^, ^13^, ^14^, ^17^ Some of these authors suggest the following causes of the increase in latency: the longer time CI users need to process auditory information^7^, ^9^, ^10^, ^11^, ^17^; post-CI functional auditory performance, suggesting a cortical reorganization in them, which is not seen in those with a moderate performance^12^ discrimination difficulties in CI users^14^; CI experience time^16^; and association with speech perception tests, presenting lower P3 component latency in the group with the worst speech perception results.^15^

Given the possibility of reestablishing auditory input with CI in people with postlingual deafness, this study aimed to verify possible functional changes in the central auditory system after the intervention, at three different moments; to correlate P3 component latency measures with the time of hearing loss, time of auditory deprivation, and speech perception test; and to associate P3 component measures with age, sex, and stimuli presented (pure-tone and speech).

## Methods

This study was approved by the institutional review board of a public hospital of a research and teaching center (protocol number 16014/2014F). It is an observational, descriptive, longitudinal, comparative study, single-subject experimental design because it is the most stable condition of the P300 test, with emphasis on diagnostic research. All participants read, accepted, and signed the informed consent form.

The inclusion criteria were individuals aged 18 years or more, of both sexes, with post lingual bilateral severe to profound sensorineural hearing loss, with indication to cochlear implantation according both the criteria established by the service's medical team and to the country's public regulations issued by the Ministry of Health on December 18, 2014, regulation number 2776.^18^

Subjects who could not discriminate the stimuli in the P300 test in any of the three phases even after training, and subjects who had problems with their CI and did not effectively use them in the 6 months after CI activation were excluded.

The sample consisted of 21 individuals aged 23–77 years. Since the literature has shown P3 component latency variations after 45 years of age,^19^, ^20^ the participants were divided into two groups for age analysis: one group under 45 years old and the other, 45 years old and over.

Minimum level responses found for phases in which the subject was using an electronic device, either Hearing Aid (HA) or CI, were registered and researched in free field. For all phases, the equipment was calibrated as dBHL.

P300 was registered in three steps: preoperative (Phase 1), at CI activation (Phase 2), and after 6 months effectively using the CI's external device (Phase 3).

For P300 registration on the first phase (preoperative), the subject was instructed to maintain frequent HA use, with the usual program and volume settings. For the two post-CI steps (activation and 6 months after activation), P300 was measured with bimodal fitting (CI in one ear and HA in the contralateral ear), both with their usual program settings.

Before exam recording, all participants were trained with rare and frequent speech and pure-tone stimuli presentation. They were asked to raise their index finger when they heard a rare stimulus. We considered the registration with a 90% correct answer rate (45 out of 50 presentations) to avoid inattention interference.

We used the two-channel Navigator Pro equipment, Bio-logic Systems Corp. Auditory Evoked Potential (AEP) System, version 1.3.0, Natus Medical, USA, connected to a conventional computer, in free field, with a loudspeaker positioned at 0° azimuth, at ear height, 80 cm away from the subject in the seated position.

For P300 electrophysiological testing, we used the approach suggested by Jasper (1958).^21^ The active electrode was positioned at Cz and connected to input 1 in the preamplifier channels 1 and 2 (jumper). Reference electrodes were placed on the left (A1) and right (A2) earlobes and connected to input 2 in the preamplifier channels 1 and 2, respectively. The ground electrode was positioned at Fpz, in the center of the forehead. We ensured that the impedance of each electrode was lower than 5 KΩ and between pairs, lower than 3 KΩ. The subject was seated at 45°, in a quiet room, with both eyes fixed at a predetermined point to avoid electrical artifacts created by eye movements and blinking during P300 registration. Component marking followed the criteria established by Junqueira and Colafêmina (2002).^22^

The general parameters used to obtain P300 latency and amplitude are described in Table 1.

**Table 1:** General parameters to obtain P300 registrations.

Stimulus		
Type	Tone-burst	Speech
Frequency	1000 Hz – frequent	/ba/ – frequent
	2000 Hz – rare	/da/ – rare
Filter	30.0 (high frequencies) and 1.0 (low frequencies)	
Gain	50,000	
Intensity	30 dBSL	
Time of analysis	512	
Stimulator	Free field	
Polarity	Alternating	
Sample	80% – frequent (approximately 200)	
	20% – rare stimuli (approximately 50)	
Task		
Attentive	Raise index finger	
Acquisition		
Channels	2 channels	
Electrodes	Cz/A1 and Cz/A2 and Fpz (ground)	
Sampling	250–300	

dBSL, decibel sensation level; Hz, hertz.

We chose to study P3 component because it is the endogenous component and reflects the brain functioning when stimulated, without being influenced by sound intensity.

### Statistical analysis

The collected data were entered into Microsoft Excel (2010) spreadsheets for proper information storage. Qualitative results were presented as frequency and proportion. Quantitative results were presented as mean and standard deviation (mean ± SD), except for the following variables: auditory threshold (dBHL), latency (ms), and amplitude (µV), which were presented as mean and Standard Error (SE). The subgroups’ ages were compared with Student's *t*-test, whereas sex was compared with the Chi-Squared test. We also used analysis of variance with repeated measures (ANOVA-RM) to compare differences in threshold (dBHL), latency (ms), and amplitude (µV) between the study's three phases (Pre-CI, at CI activation, and after 6 months of CI use) combined with the following factors: sex (M-male vs. F-female), age (<45 vs. ≥45), stimuli (speech vs. pure-tone). The Tukey HSD test confirmed differences between multiple comparisons. Pearson's correlation test was used to correlate WRS (Word Recognition Score) (%) with the threshold (dBHL).

All procedures were processed with the JMP® 10.0 software (SAS Institute Inc., Cary, NC, USA). The significance of *p* < 0.05 was used.

## Results

A total of 21 individuals met the inclusion criteria. They were 23–77 years old (mean 48.8 years, SD ± 16.3), of both sexes, nine male (42.9%) and 12 female (57.1%).

There was a prevalence (n = 12, 57.1%) of right-sided implantations. Eleven subjects (52.4%) were implanted with CIs manufactured by Oticon Medical®, 5 (23.8%) with Advanced Bionics®, 4 (19%) with Cochlear®, and 1 (4.8%) with Med-El®. Hearing loss etiology is still under investigation for most subjects (61.9%).

The participants’ mean auditory threshold (500, 1000, 2000, and 4000 Hz) was 112.5 dBHL (SD ± 13.6). Results for Audiometric Threshold (AT) gain were better with bimodal fitting – already noted at CI activation (mean AT = 49.4 dBHL, SD ± 10.1) – than with bilateral HAs (mean AT = 73.7 dBHL, SD ± 12.9).

In the mean AT, there was a difference of 38.8 dBHL between those with and without Has, and of 63.1 dBHL with bimodal fitting (CI in one ear and HA in the contralateral ear) at CI activation (*p* < 0.0001) – which shows an even greater improvement, with a difference of 74.3 dBHL 6 months after CI activation, with a gain of up to 11.2 dBHL for the last phase compared with activation (Table 2).

**Table 2:** Audiological data from the subjects obtained in free field (n = 21).

	Mean AT (500 Hz, and 1, 2, and 4 kHz)			WRS with HA	WRS with CI
	HA (RE + LE)	CI activation	6 m after CI	Pre-CI	6 m after CI
Subject	(dBHL)	(dBHL)	(dBHL)	(%)	(%)
1	77.5	48.8	25.0	28	72
2	58.1	47.5	48.8	60	88
3	57.5	48.8	38.8	80	80
4	58.8	31.3	26.3	96	100
5	75	50.0	35.0	44	84
6	79.4	43.8	32.5	0	92
7	93.1	41.3	41.3	0	64
8	55	28.8	31.3	92	100
9	93.1	52.5	27.5	0	80
10	82.5	53.8	41.3	4	60
11	68.8	55.0	48.8	52	92
12	81.9	33.8	28.8	0	52
13	90	42.5	31.3	0	72
14	52.5	51.3	37.5	16	68
15	81.9	51.3	37.5	44	96
16	65	68.8	48.8	0	92
17	65.6	55.0	42.5	0	68
18	82.5	66.3	41.3	0	–
19	83.8	55.0	46.3	0	84
20	–	52.5	52.5	0	76
21	72.5	58.8	40.0	28	96
Mean	73.7	49.4	38.2	25.9	80.8
SD	12.9	10.1	8.1	33.2	14.0

AT, auditory thresholds; CI, cochlear implant; dBHL, decibel hearing level; HA, hearing aid; RE, right ear; LE, left ear; WRS, word recognition score (trisyllabic words); m, months.

It is worth noting that, in the free field evaluations, the results correspond to the better ear – i.e., in this study, the CI ear, since our sample consists of people with symmetrical profound hearing loss, except for one subject with severe hearing loss.^23^

The results for P3 component latency measures with pure-tone and speech stimuli are shown in Table 3.

**Table 3:** Mean P3 latency measures in the three study phases (pre-CI, at CI activation, and six months after CI), in derivations Cz/A1 and Cz/A2, with tone-burst and speech stimuli (n = 21).

Stimuli	Phases	Cz/A1 latency (ms)		Cz/A2 latency (ms)		ANOVA results (*p*-value)		Tukey HSD test*
		Mean	SE	Mean	SE	Cz/A1 vs. Cz/A2	Phases	(*p*-Value)
Tone-burst	Pre-CI (Phase 1)	352.9	10.4	349.3	10.7	0.4947	0.0275	Phase 2 vs. Phase 3 0.0211
	CI activation (Phase 2)	364.9	11.4	364.8	11.1			
	Six months after CI (Phase 3)	336.2	9.8	335.8	10.3			
Speech	Pre-CI (Phase 1)	321.9	12.1	319.3	12.6	0.9530	0.0368	Phase 1 vs. Phase 2 0.0359
	CI activation (Phase 2)	368.7	15.0	367.5	15.5			
	6 months after CI (Phase 3)	343.6	13.5	341.8	9.8			

Ms, milliseconds; SE, standard error; vs., versus; CI, cochlear implant.

* Tukey HSD test, significant *p*-value (<0.05).

There was no statistical evidence in the comparison of latencies for derivations Cz/A1 and Cz/A2, with pure-tone (*p* = 0.4947) and speech (*p* = 0.9530) stimuli.

Different data collection phases (Phase 1: Pre-CI, Phase 2: at CI activation, and Phase 3: after 6 months of CI use) we verified statistically significant differences for both pure-tone (*p* = 0.0275) and speech (*p* = 0.0368) stimuli. It was identified for pure-tone when Phases 2 and 3 were compared (*p* = 0.0211) and for speech stimuli when Phases 1 and 2 were compared (*p* = 0.0359), both with an increased P3 latency during Phase 2 (Table 4).

**Table 4:** Analysis of P3 latency (ms) and amplitude (µV) measures in three phases (Pre-CI, at CI activation, and six months after CI), with sex and age, with tone-burst stimulus.

Phases	Sex				Age					
	F		M		<45 years		≥45 years			
	Mean	SE	Mean	SE	Mean	SE	Mean	SE	ANOVA	Tukey test
Pre-CI (Phase 1)	342.07	15.10	356.14	13.84	335.84	13.65	366.40	13.71	Latency – *p* = 0.0314	
	4.85	0.80	5.00	0.93	5.72	1.03	4.18	0.72		
CI activation (Phase 2)	369.82	17.32	358.83	13.86	332.98	12.94	390.97	13.04	Amplitude – *p* = 0.1679	Latency – *p* = 0.0248 Phase 2 vs. Phase 3
	4.51	0.75	3.40	0.58	4.93	0.83	3.26	0.51		
6 months after CI (Phase 3)	327.14	12.12	346.30	17.25	305.13	11.66	356.18	12.08		
	5.14	0.61	5.91	0.87	4.81	0.67	5.94	0.71		
ANOVA (*p*-value)	Latency – *p* = 0.8567				Latency – *p* = 0.0054					
	Amplitude – *p* = 0.5814				Amplitude – *p* = 0.2280					

ms, milliseconds; µV, microvolt; F, female; M, male; SE, standard error; vs., versus; CI, cochlear implant (significant *p*-value <0.05).

No significant differences were found for either stimulus when Phases 1 and 3 were compared.

No statistical differences were found in amplitude measures for derivations Cz/A1 and Cz/A2, using pure-tone (*p* = 0.8721) and speech (*p* = 0.9388) stimuli, neither between the three phases with pure-tone (*p* = 0.1779) and speech (*p* = 0.3876) stimuli. Nevertheless, we have observed a trend of amplitude increase with both stimuli after 6 months of CI use.

**Figure 1:**
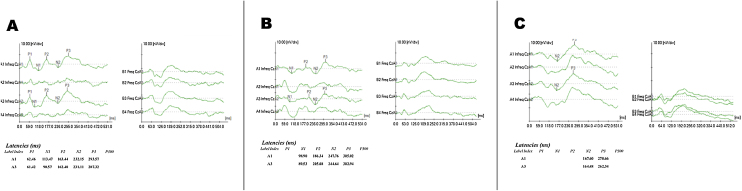
Example of P300 recording with tone-burst stimulus in three phases, A, Phase 1 (Pre-CI); B, Phase 2 (at CI activation); C, Phase 3 (6 months after CI).

**Figure 2:**
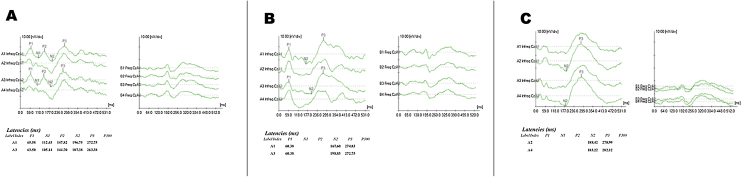
Example of P300 recording with speech stimulus in three phases, A, Phase 1 (Pre-CI); B, Phase 2 (at CI activation); C, Phase 3 (6 months after CI).

Figure 1, Figure 2 show examples of exam recordings.

Since there was no statistical difference when we compared latency for derivations Cz/A1 and Cz/A2 (Table 4, Table 5), we verified associations between P3 component latency measures and the different stimuli in the three phases, regarding sex and age, we used mean derivations Cz/A1 and Cz/A2.

**Table 5:** Analysis of P3 latency (ms) and amplitude (µV) measures, in three phases (pre-CI, CI activation, and six months after CI), with sex and age, with speech stimulus.

Phases	Sex				Age					
	F		M		<45		≥45		ANOVA	Tukey test
	Mean	SE	Mean	SE	Mean	SE	Mean	SE	*p*-Value	*p-*Value
Pre-CI (Phase 1)	321.26	25.56	318.46	12.20	300.51	17.52	342.81	13.15	Latency – *p* = 0.0351	Latency
	7.19	1.35	3.70	0.68	5.81	1.05	4.66	1.52		
CI activation (Phase 2)	341.23	18.64	406.48	17.20	341.86	26.42	391.42	12.76	Amplitude – *p* = 0.089	Phase 1 vs. Phase 2 – *p* = 0.0253
	5.26	0.81	3.44	0.77	4.74	1.11	4.30	0.62		
6 months after CI (Phase 3)	329.82	12.19	357.46	18.11	313.77	12.69	362.03	12.77		Phase 2 vs. Phase 3 – *p* = 0.0347
	5.17	1.42	6.36	0.74	6.59	1.77	5.15	0.74		
ANOVA (*p*-value)	Latency – *p* = 0.3215				Latency – *p* = 0.0117					
	Amplitude – *p* = 0.1880				Amplitude – *p* = 0.9670					

ms, milliseconds; µV, microvolt; F, female; M, male; SE, standard error; vs., versus; CI, cochlear implant (significant *p*-value <0.05).

For pure-tone stimulus, there was an increased P3 latency when comparing the different age ranges (*p* = 0.0054), with higher latencies in individuals ≥45 years old. There was no difference in latency when sex was compared (*p* = 0.8567). There was a significant decrease in mean latency values when Phases 2 and 3 were compared (*p* = 0.0248). There was no difference in mean P3 component latency values when comparing Phase 1 with 2 and Phase 1 with 3 – although values were higher in Phase 2 than in Phase 1, except for individuals under 45 years old (Table 4).

The associations between P3 component latency measures with speech stimulus and sex and age in the three phases are shown in Table 5. We can see, once again, that when speech was used, latency means were not associated with sex (*p* = 0.3215). On the other hand, when associated with the different age ranges, there was a statistical difference (*p* = 0.0117).

In the association of latency with speech stimulus in the three phases, we found a significant difference comparing Phases 1 and 2 (*p* = 0.0253) and Phases 2 and 3 (*p* = 0.0347), whereas latency increase was noticed in Phase 2 (Table 5). There was no significant difference between Phases 1 and 3 (*p* = 0.6198).

No associations were found between P3 component amplitude measures and the different stimuli (pure-tone and speech) in any of the three phases (*p* = 0.1679 and *p* = 0.089) when we analyzed sex (*p* = 0.5814 and *p* = 0.1880) and age (*p* = 0.2280 and *p* = 0.9670).

There was no statistical difference for latency (*p* = 0.6935) or amplitude (*p* = 0.4694) when pure-tone was compared with speech stimulus for P3 component registration in the three phases (ANOVA).

**Figure 3:**
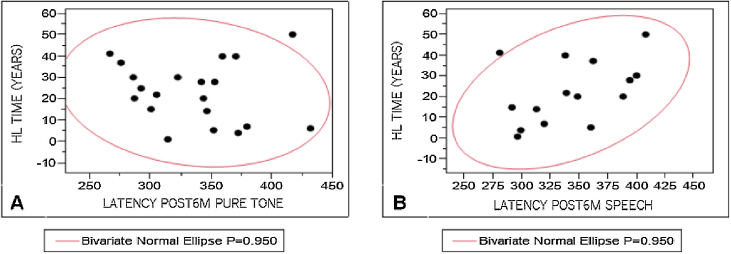
Correlation between P3 latency measures and time of hearing loss (HL time), after six months of CI, with tone-burst (A, r = −0.146) and speech (B, r = 0.438522).

To analyze the influence of the variables studied on P300 registration, P3 component latency measures were correlated with WRS results, hearing loss characteristics, time of hearing loss, and time of auditory deprivation. The results were divided according to the stimulus used to elicit a response. Pearson's test was used for these correlations, considering 0.4–0.5 as a weak correlation, 0.5–0.7, a moderate correlation, and above 0.9, a strong correlation. A weak correlation was observed (Fig. 3) only between P3 component mean latency levels with speech stimulus and time of hearing loss (r = 0.44).

## Discussion

Despite the age range in the sample, P300 data collection allowed us to carry out an intragroup comparison in the different phases.

The latency values obtained in this study are similar to those found in the literature for normal hearing individuals. Depending on the phase, P3 component, they ranged from 336.2 to 364.9 ms with pure-tone,^1^, ^24^, ^25^, ^26^, ^27^ and from 321.9 to 368.7 ms with speech.^5^, ^8^, ^9^, ^10^, ^11^, ^17^, ^28^ Also, there was no significant difference in amplitude and latency between the groups.^5^ However, authors found increased latency in CI users when compared with normal hearing subjects, and they associated auditory processing ability to P3 component latency measures in CI users, which means faster auditory processing is connected to better speech recognition ability in CI users.^11^ Other authors considered P300 to be a neural predictive biomarker, sensitive to cortical processing, and capable of providing information about sound reception and neural codification of different acoustic-phonetic cues. In CI users, processing time can be prolonged, depending on intrinsic aspects and top-down processing.^9^

Mean P3 component latencies in the three phases, using both pure-tone and speech, did not vary for derivations Cz/A1 and Cz/A2. In the interphase comparison, there was a difference between Phases 2 and 3 for pure-tone and between Phases 1 and 2 for speech. We observed a higher latency in the CI activation phase, with a significant difference for speech and a trend of increase for the pure-tone. Also, individuals who did not elicit a P3 wave during Phase 1 did so at activation, with increased latency – the pure-tone was used in six subjects and speech, in five subjects. The fact that these individuals could not detect rare stimuli in the Pre-CI phase and then managed to do it at activation, even with increased latency, might be related to the time of auditory deprivation – i.e., insufficient auditory stimulation for the individual to properly discriminate rare stimulus. Such ability progressively improves as central auditory system stimulation is reestablished, which can reflect on neuroplasticity.

Increased P3 latency at the CI activation phase occurred for both pure-tone and speech stimuli (Table 4, Table 5), with a higher latency for speech stimulus. This can be related both to the adjustment of the CI speech processor and, especially, to the greater complexity of speech, which requires more comprehension and attention. It is worth noting that P3 component latency corresponds to the time and speed needed for stimulus processing. Therefore, it is a temporal resolution measurement, which may be influenced by attention, memory, and auditory processing.^29^ Increased latency in CI users was also noted by Okusa et al. (1999)^14^ when the pure-tone was used and when rare stimulus frequency got closer to frequent stimulus frequency – possibly because CI provides less efficient pitch discrimination than the cochlea.

Few authors have discussed P3 component amplitude changes or variations. These authors report decreased P3 amplitude related to higher difficulty in stimulus discrimination,^10^, ^13^, ^30^ to stimulus complexity,^5^, ^8^ or to attentional resources needed for the task in proportion with the working memory.^10^, ^29^, ^31^ This reinforces the idea that CI use time associated with auditory training may influence P3 component latency and amplitude measures as a positive response to intervention monitoring.^14^

The age groups were divided at 45 years old to avoid interferences in the study. As we divided subjects into different age ranges, pure-tone revealed a significant difference for P3 component latency in individuals over 45 years old (*p* = 0.0054) (Table 4). Studies in normal hearing individuals already present increased P3 component latency in individuals older than 45 years.^20^ Authors have also identified a linear latency increase of 2.85 ms every five years in older subjects^19^ due to cognitive decline inherent to aging.

We analyzed the influence of variables on P3 component latency measures and found a weak correlation between P3 component mean latency with speech stimulus and time of hearing loss. This happens possibly because the CI user needs to make a greater effort to listen, improving their results after some time of use (Phase 3). Similar results were found by Beynon et al. (2005),^8^ who compared normal hearing individuals with CI users, verifying that people who had used the CI for 2–4 years had higher latency and lower amplitude measures than the control group.

Besides attention, other cognitive abilities are related to P300. We did not analyze working memory in this study because the task did not require the subject to continuously count rare stimuli. However, the executive cognitive function, which is directly connected to the task used in P300, requires action planning and execution that correspond in this study to raising the index finger in the presence of a rare stimulus.

The relationship between increased P3 component latency and individuals with low scores in speech perception tests has been detected by Kubo et al. (2001)^15^ and Iwaki et al. (2004)^32^ using pure-tone stimulus. Groenen et al. (2001)^33^ and Makhdoum et al. (1998)^31^ found results similar to those in our study, that is, no difference between, speech and pure-tone stimuli. On the other hand, they reported correlations between amplitude measures and speech perception.

There is a tendency in the literature to associate electrophysiological tests with imaging tests to study possible cortical changes involving auditory abilities, to both reach a diagnosis and monitor intervention. The literature has highlighted the need to minimize the impact of auditory sensory deprivation on cognition.^34^, ^35^, ^36^, ^37^ The study by Lévesque, Théoret, and Champoux (2014),^38^ who found a positive correlation between auditory deprivation and cognitive task difficulties, has also emphasized it. This is confirmed by Stropahl et al. (2017),^39^ who reported behavioral, electrophysiological, and neuroimaging evidence suggesting that new patterns of cortical activation may identify signs of neuroplasticity in individuals who received electrical stimulation with CI after a period of auditory deprivation.

## Conclusions

There were changes in P3 component latency during the period assessed, for both speech and pure-tone stimuli, with increased latency in the activation phase and similar lower results in the two other phases, Pre-CI and 6 months after CI use. Mean amplitude measures did not vary in the three phases.

## Funding

10.13039/501100002322CAPES (Coordination for the Improvement of Higher Education Personnel).

## Ethical approval

The study was approved by the Ethics Committee of the University Hospital, Medical School of Ribeirão Preto and registered at the Clinical Trials Program (NCT03352154).

## Conflicts of interest

The authors declare no conflicts of interest.
